# Flexural Behavior of Reinforced Concrete Two-Way Slabs Strengthened with Basalt Fiber-Reinforced Polymer Grid and Engineered Cementitious Composite

**DOI:** 10.3390/ma19102019

**Published:** 2026-05-13

**Authors:** Jifeng Xue, Mingyu Zhu, Hongjun Liang, Haoyu Li

**Affiliations:** 1 School of Civil Engineering, Nantong Institute of Technology, Nantong 226000, China; xjf_2007@126.com; 2 School of Civil Engineering, Wuhan University, Wuhan 430072, China; 2024102100038@whu.edu.cn (M.Z.); 2023202100040@whu.edu.cn (H.L.)

**Keywords:** RC two-way slabs, BFRP grid, ECC, flexural behavior

## Abstract

**Highlights:**

- The BFRP grid–ECC composite increased the ultimate slab capacity by 154.1–236.1%, demonstrating an effective epoxy-free CRM retrofitting solution for slabs.
- Failure shifted from continuous diagonal cracks to fine, discontinuous short cracks, indicating that the composite layer effectively suppresses crack propagation and enhances ductility.
- Thicker BFRP grids and ECC layers consistently improved flexural load and stiffness, suggesting that appropriately increasing CRM thickness is conducive to reinforcement.
- Y-shaped and triangular yield line models accurately predicted the ultimate capacity, providing a reliable design basis for field applications.

**Abstract:**

This paper innovatively employs an epoxy-free composite layer with basalt fiber-reinforced polymer (BFRP) and engineered cementitious composite (ECC) to reinforce the two-way concrete slab structure. Five strengthened slabs and one reference slab were tested under biaxial bending moments with four-side simply supported conditions. The thickness of ECC (15, 25, 35 mm) and BFRP grid (1, 2, 3 mm) were selected as two main variables in the test program. The experimental results showed that the cracking and ultimate load of the strengthened slabs were substantially improved. Notably, the cracking pattern was shifted from diagonally concentrated cracks to discontinuous short cracks, with no apparent debonding of the composite layer. As the thickness of the BFRP grid and ECC increases, both the flexural capacity and stiffness improve, with decrease in the maximum deflection and effective utilization rate of steel reinforcement and BFRP grid at mid-span. Furthermore, a theoretical model considering different positional distribution of yield line was proposed to predict the bearing capacity of the strengthened slabs, with the calculated values aligned well with the experimental results. This research highlights the FRP–ECC composite as a robust reinforcement method for two-way slabs, and offers a good design-oriented reference basis in the field.

## 1. Introduction

Urban renewal in medium-to-large cities encounters challenges with deteriorating RC structures affected by corrosion, earthquakes, and fires. Demolition and reconstruction, while often necessary, pose environmental risks. It is imperative to implement strengthening and retrofitting measures to enhance the load-bearing capacity of deficient structures [1,2,3]. Fiber-reinforced polymer (FRP), known for its corrosion resistance, high strength, and lightweight properties, is increasingly favored as a material for enhancing existing weakened structures [4,5].

The versatility of FRP allows for fabrication into various forms such as various FRP profiles and grids. FRP can be applied to aging RC structures through different techniques [6,7]. Conventional methods involve utilizing organic polymers like epoxy and polyester as matrices and adhesives for externally bonding (EB) FRP to concrete surfaces [8,9]. However, the durability of organic polymers under environmental conditions and their bonding efficacy with concrete fall short of engineering requirements [10,11,12]. The near-surface-mounted (NSM) technique has emerged as an alternative to improve FRP’s bonding with concrete, ensuring reliable adhesion and enhancing the utilization efficiency of FRP materials [13,14]. Despite the advantages of the NSM technique, its application is limited due to the complexity of groove fabrication and precise installation requirements. A novel composite-reinforced mortar (CRM) layer, comprising FRP and inorganic mortar, is gaining traction for strengthening deficient RC flexural members, owing to its ease of construction, exceptional durability, and compatibility with concrete [15,16,17,18].

The efficacy of the CRM strengthening technique in enhancing the flexural behavior of RC structures has been investigated in several experimental studies. Notably, ref. [19] explored the substitution of cementitious materials for epoxy as bonding agents, resulting in improved composite action and minor adjustments in design procedures. Ref. [20] compared the effectiveness of several BFRP-based strengthening methods on one-way RC slab, highlighting the advantages of using CRM in terms of reduced crack width and increased load-carrying capacity. Ref. [21] used CRM, composed of GFRP grids and mortar, to strengthen pre-stressed RC slabs lightened with hollow clay elements, demonstrating its effectiveness in enhancing structural integrity. However, the cement-based nature raises concerns over carbon emissions and embodied energy. As a low-carbon alternative, engineered geopolymer composites (EGCs) have been developed using industrial by-products, significantly reducing environmental footprint. Recent studies on BFRP grid-reinforced geopolymer mortar for tunnel linings [22] and basalt fiber–engineered geopolymer composites for fire-damaged columns [23] demonstrate the feasibility of such sustainable strengthening systems.

Among other materials, ECC, recognized for its strain-hardening behavior and large tensile strain capacity, stands out as a promising mortar for the CRM flexural strengthening method [24,25,26]. ECC’s notable advantage lies in its strain-hardening behavior under tension, boasting a substantial tensile strain capacity that proves beneficial whether used independently or in combination with FRP for flexural reinforcement [27,28]. Previous studies have confirmed that the FRP grid–ECC composite exhibits higher ultimate stress levels than pure ECC [29]. This composite layer enhances stress distribution uniformity at the interface between the FRP and substrate concrete, thereby delaying deformation and debonding of FRP systems [30]. Leveraging ECC’s durability, strong concrete bonding, and tensile deformation capacity, the FRP–ECC composite strengthening technique emerges as a highly effective solution for structural reinforcement [31].

In their research, ref. [32] strengthened RC beams with FRP–ECC composites. A comparison with traditionally EB FRP-strengthened beams revealed that the addition of an ECC layer altered the behavior of the CFRP strip, transitioning it from debonding to rupture at the ultimate state. This change significantly increased the flexural capacity, energy consumption capacity, and ductility of the beams. Studies by Refs. [33,34] highlighted ECC as an optimal material for enhancing the flexural behavior of FRP grid/bar reinforced RC beams, with the CFRP–ECC system offering improved ductility due to multiple cracking behavior in the ECC matrix, facilitating more uniform shear stress transfer at the interface between the strengthening layer and the substrate concrete. In particular, ref. [34] emphasized the necessity of reliable end anchorage for this strengthening system. Ref. [35] tested the CFRP–ECC hybrid strengthening method on RC beams under four-point bending, observing premature failure due to CFRP–ECC debonding, again emphasizing the importance of secure anchorages. Conversely, experiments by Refs. [36,37] demonstrated good bonding nearly until the final stages of damage in strengthened beams. FRP–ECC, a typical and promising candidate for strengthening deficient RC members using CRM, has been effectively employed in the flexural and shear strengthening of RC beams [38,39]. While CRM is rigorously defined by EAD 340392-00-0104 [40] and widely guided by ACI 549.4R-20 [41] and CNR-DT 215/2018 [42], its application has predominantly focused on masonry or one-way members. A significant research gap remains when extending this technique to RC two-way slabs with more complex stress states. The existing literature predominantly focuses on the reinforcement mechanisms under uniaxial stress, overlooking the influence of intricate biaxial stress fields on the interfacial shear transfer between the strengthening layer and the concrete substrate. Furthermore, the continued reliance on organic epoxy-based adhesives in many systems poses durability concerns and often leads to premature intermediate crack-induced debonding. To date, there is a lack of systematic evaluation regarding the synergistic response of epoxy-free FRP grid–ECC systems in two-way slabs, particularly their ability to suppress biaxial cracking. Thus, the design and extension of reinforcement using FRP grid-reinforced ECC composite layers in two-way slabs remains insufficiently explored and requires further investigation.

To address these deficiencies, this study systematically investigates the flexural behavior of RC two-way slabs strengthened with a BFRP grid–ECC composite layer for the first time. The novelty of this research is three-fold: First, an entirely epoxy-free inorganic strengthening system is employed, achieving good bond via substrate, simply roughening and eliminating the debonding issues inherent in traditional methods. Second, through an in-depth analysis of crack evolution and failure modes under biaxial bending, this work elucidates the internal mechanism by which the strain-hardening properties of ECC transform crack patterns and enhance global stiffness. Finally, this paper proposes a novel analytical model that accounts for three different yield line patterns, providing a more rigorous and accurate design basis for two-way slabs retrofitting than existing linear models.

## 2. Experimental Program

### 2.1. Specimen Details

**Figure 1:**
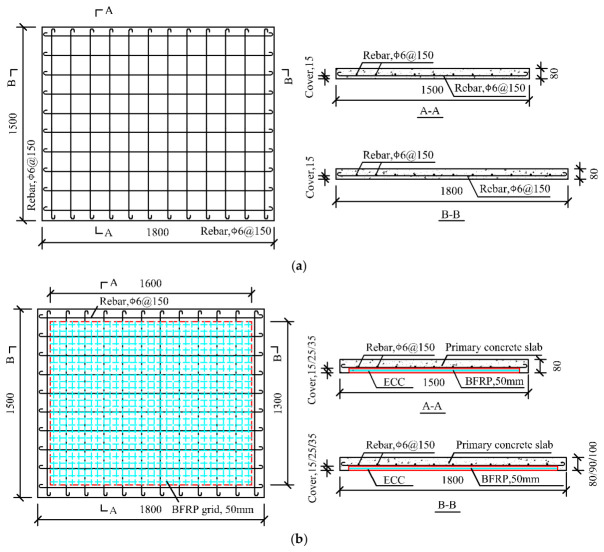
Geometry of tested slabs. (**a**) Reference slab; (**b**) BFRP grid–ECC strengthened slab.

Six RC two-way slabs were constructed, including five strengthened slabs and one traditional RC slab for reference. Previous studies confirm a suitable BFRP grid thickness of 1–5 mm [43]. The basic thickness of ECC is set equal to the cover thickness of the old concrete slab. Per GB 50010-2010 [44], the minimum concrete cover in ordinary RC slabs is 15 mm. The study variables included BFRP grid thicknesses (1 mm, 2 mm, and 3 mm) and ECC thickness (15 mm, 25 mm, and 35 mm). All slabs shared dimensions of 1500 mm × 1800 mm × 80 mm, as illustrated in Figure 1. The reference slab, T-0-0, had a 15 mm concrete cover over the steel reinforcement. The other five slabs were reinforced with a CRM layer of BFRP grid and ECC. Each strengthened specimen was denoted by letters and numbers, where letters T indicated two-way slabs, F (BFRP grid) or E (ECC) represented strengthening materials, and numbers indicated material thicknesses. For example, specimen T-F1-E25 denoted a two-way slab strengthened by 1 mm BFRP grid and 25 mm ECC, with details outlined in Table 1. Longitudinal and transverse steel reinforcement was arranged at 150 mm intervals, forming a mesh framework measuring 1800 mm × 1500 mm. The BFRP grids, sized at 1600 mm × 1300 mm, had 50 mm × 50 mm square openings.

### 2.2. Materials

The six RC slabs were cast from a uniform concrete batch of ordinary Portland cement concrete (OPC) with a compressive strength grade of C25. Testing three standard cubic samples yielded an average 28-day compressive strength of 26.3 MPa. In addition, Table 2 summarizes the statistical data of the strength indices of each material. It can be observed that the material strengths exhibit relatively low variability, indicating that the material quality meets the requirements.

HPB 300 steel bars (made of ordinary low-carbon steel), each 6 mm in diameter, were utilized with an average yield strength of 353 MPa and ultimate tensile strength of 514 MPa.

BFRP grids, sourced from Jiangsu Green Materials Valley New Material T and D Co., Ltd., Nanjing, China, had a mesh size of 50 mm × 50 mm and thicknesses of 1 mm, 2 mm, and 3 mm. The mechanical properties of the BFRP grid were determined through three tensile tests as per JC/T 2461-2018 [45], and are detailed in Table 2 and Table 3. The use of BFRP materials is mainly considered to balance economy and performance.

**Figure 2:**
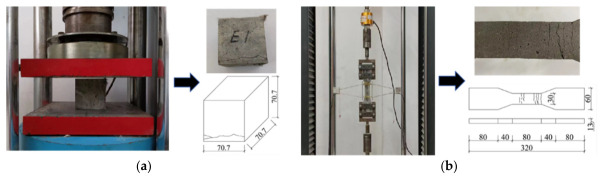
Compressive and tensile coupons and tests of ECC. (**a**) Compressive test; (**b**) tensile test.

The ECC mix comprised ordinary Portland cement (P.O 42.5), water, silica sand (80–120 mesh), fly ash (5000 mesh), and a polycarboxylate-based water reducer in a ratio of 1: 0.725: 1.044: 1.9: 0.0116. PVA fibers, added at a 2% volume fraction in the ECC, enhanced tensile deformation of ECC by facilitating a bridging effect. The utilized PVA fibers featured a length measuring 12 mm, a diameter of 40 µm, and a density at 1.3 g/cm^3^. The tensile strength, Young’s modulus, and elongation of PVA fibers were 1556 MPa, 36.2 GPa, and 7.7%, respectively. The average 28-day ECC compressive strength, determined from testing three standard cubic samples, was 37.23 MPa. Compression failures revealed a primary crack perpendicular to the loading direction (see Figure 2a). According to JC/T 2461-2018 [46], testing four dumbbell-shaped ECC samples indicated an average 28-day ECC ultimate tensile strength of 3.77 MPa and an elongation of 2.62%. Tension failure exhibited multiple parallel cracks, as shown in Figure 2b, demonstrating characteristic strain-hardening behavior.

### 2.3. Preparation of Strengthened Specimens

**Figure 3:**
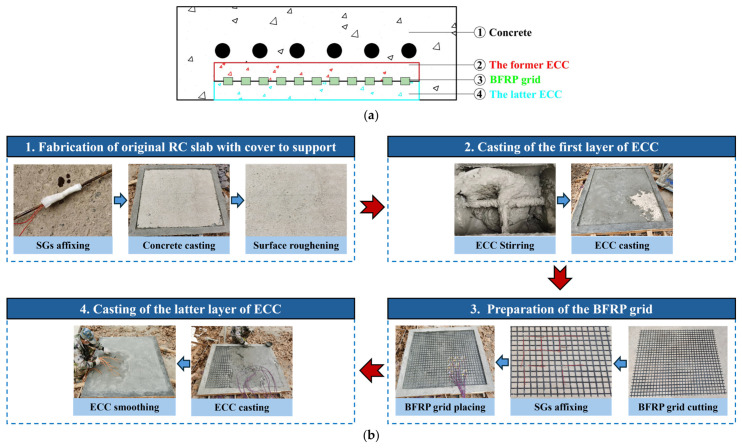
Fabrication procedure of BFRP grid–ECC strengthened slab. (**a**) Section view of the strengthened slab; (**b**) specific fabrication procedure.

The experiment focuses on replacing damaged concrete in the inner support ring with a BFRP grid–ECC composite layer while preserving the cover above the support during the strengthened slab fabrication. Figure 3 shows the manufacturing sequence of the strengthened slabs through the area marks of different colors. Taking specimen T-F1-E25 as an example, the specific steps are as follows:

1. Fabrication of original RC slab with cover to support. First, construct and fix wooden formworks of size 1800 mm × 1500 mm × 80 mm and 1600 mm × 1300 mm × 15 mm. Affix strain gauges to the pre-fabricated rebars with adhesives, protecting them with silicone and epoxy resin-soaked gauze. Rebars are tied into a mesh using binding wire, and place it on the 1600 mm × 1300 mm × 15 mm wooden formwork. Next, pour concrete, compact it with a vibrator, level the surface, and cure it under an impermeable membrane for 28 days. Then, the bottom surface of the reinforced area was roughened for effective bonding.
2. Casting of the former layer of ECC. The prepared ECC is poured in the reinforced area, and the thickness is half of the composite layer.
3. Preparation of the BFRP grid. Cut to size, and paste strain gauges. Then, the BFRP grid is pressed gently into the fresh initial ECC, which is located in the middle of the composite layer in the thickness direction.
4. Casting of the latter layer of ECC. Pour the remaining ECC, remove the formwork 2 days later, and continue curing until 28 days.

Overall, two types of test specimens were designed for this study. One reference slab was made of concrete and steel rebar. Five strengthened slabs comprised concrete, steel rebar, ECC, and BFRP. Material mass for each specimen is given in the Table 4.

### 2.4. Test Setup

**Figure 4:**
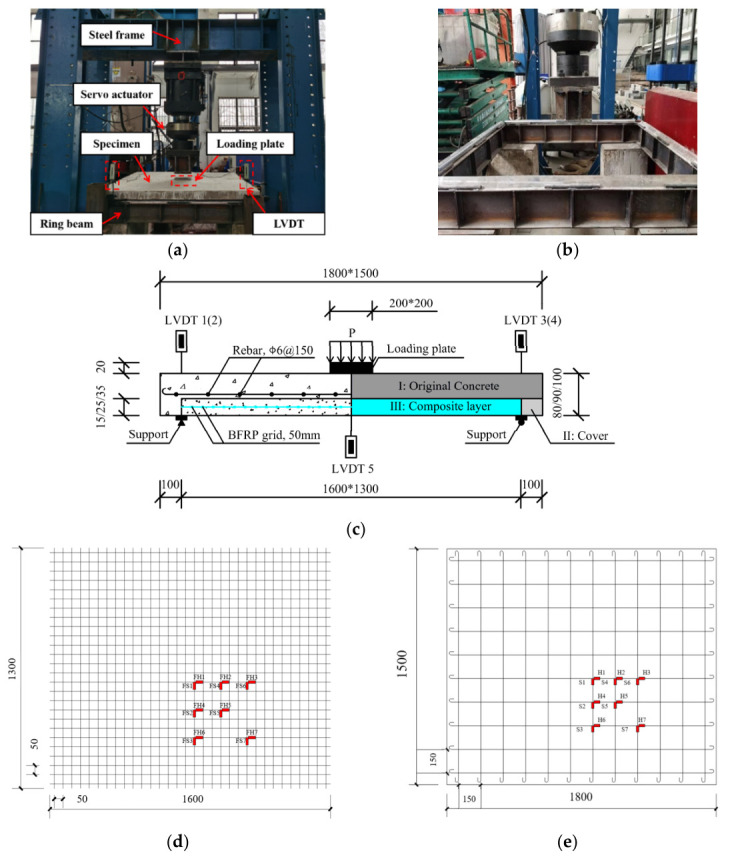
Single-point flexural tests for test slabs. (**a**) Loading instrumentations; (**b**) details of the supports; (**c**) reinforcement details of the specimen; (**d**) SG positions on BFRP grid; (**e**) SG positions on reinforcements.

All slab specimens underwent testing in a single-point bending configuration. The test setup is presented in Figure 4a. Specimens were supported on four sides, as shown in Figure 4b. The supports were situated on a ring beam with an I-shaped cross-section, anchored by four concrete piers. The entire test apparatus met the required stiffness criteria. As per GB 50152–2012 [47], the specified nominal limit state conditions for the test slab encompass:

- Failure due to the main steel reinforcement snapping in tension or exceeding a strain of 0.01.
- Concrete crushing on the compressive surface of the slab.
- Mid-span deflection surpassing 1/50 of the span (28 mm in this study).
- Maximum crack width exceeding 1.5 mm.
- Sudden failure of the BFRP grid.
- Debonding failure of the composite reinforcement layer.

The vertical load variations were recorded using a force transducer attached to the 200 t servo actuator. To measure the vertical deflections, five linear variable differential transformers (LVDTs) were utilized, one at the mid-span and four others at the corners of the test specimen, illustrated in Figure 4c. Strain gauges (SGs) with a gauge length of 3 mm were employed to track strain responses of the steel reinforcement and BFRP grids. The arrangement of SGs is demonstrated in Figure 4d,e. All slabs underwent testing with a monotonically increasing load until failure, employing displacement control at a loading rate of 0.2 mm/min, as per GB 50152-2012 [47]. The displacement loading proceeded at 0.2 mm per step until cracking occurred on the tensile surface of the specimens. Subsequently, each 2 mm increment was treated as a step following the yield of the tensile steel reinforcement. Further, every 1 mm increment was considered a step until failure. A load step was sustained for approximately 5 min before the appearance of cracks, and extended to 10 min per step post-crack initiation.

## 3. Experimental Results and Analysis

### 3.1. Failure Mode and Cracking Pattern

In this test program, all six specimens exhibited flexural failure, characterized by tensile cracks exceeding 1.5 mm at the bottom. On the other hand, the failure of all specimens was governed by the yielding of internal steel reinforcement followed by circular compressive cracks near the loading plate on the top. Two-way thin slabs are prone to punching shear failure under concentrated loads [48,49], and previous studies have also reported debonding issues using FRP grid–ECC composite [34,50]. Notably, in this study, no punching shear failure was observed in any specimen (i.e., no diagonal cracks extending through the thickness), and the BFRP grid–ECC composite layer in all strengthened specimens showed no signs of debonding. However, the progression of crack development varied between the reference and strengthened slabs.

**Figure 5:**
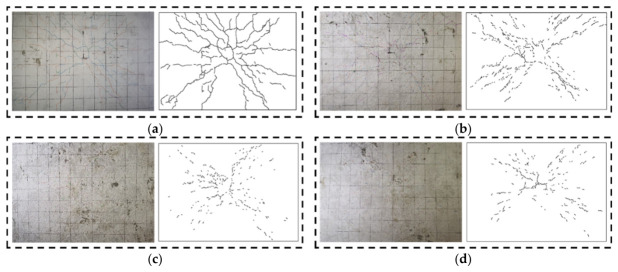

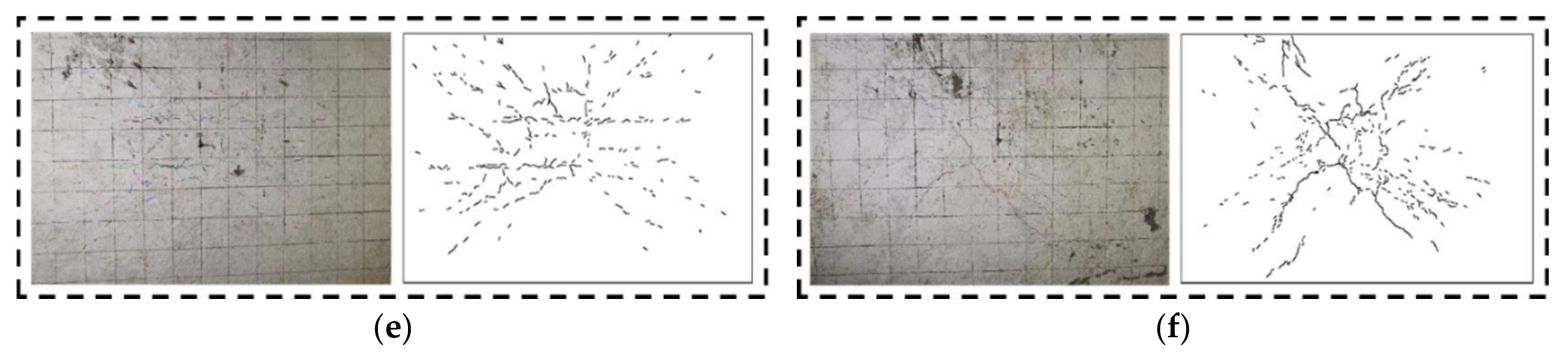
Failure mode of strengthened and reference slabs. (**a**) T-0-0; (**b**) T-F1-E25; (**c**) T-F2-E25; (**d**) T-F3-E15; (**e**) T-F3-E25; (**f**) T-F3-E35.

In the reference slab without the composite layer, the first visible crack, running parallel to the long-span direction, emerged at a load of 10 kN, measuring approximately 0.03 mm in width and 25 mm in length. Subsequent flexural cracks continued to propagate towards the span direction with increasing load. By the time the load reached 21 kN, diagonal cracks expanded significantly, causing the corners of the slab to uplift. As the load further increased to 36 kN, the maximum crack width near the loading point exceeded 1.5 mm, and all four corners visibly lifted. Upon failure, circumferential cracks encircled the loading plate on the top, while diagonal cracks extended towards the corners, leading to a series of continuous main cracks, as illustrated in Figure 5a. This typical flexural failure mode under concentrated loading has been well documented [51,52], where cracks propagate freely once initiated due to the absence of crack-bridging mechanisms.

Slab T-F1-E25 was strengthened with a 1 mm thick BFRP grid and a 25 mm thick ECC composite layer. As the load approached 27 kN, fine cracks emerged, measuring approximately 0.054 mm in width. During this period, crack development mainly involved an increase in the number of cracks, with lengths staying below 50 mm and widths less than 0.216 mm. By the time the load reached 91.5 kN, the crack width at the mid-span exceeded 1.5 mm. Upon failure, circumferential and diagonal cracks formed, akin to the reference slab. Notably, these cracks tended to be shorter, with the longest crack not surpassing 100 mm, and exhibited some spacing between them, as depicted in Figure 5b.

In the case of the remaining strengthened slabs, failure patterns were akin to those observed in slab T-F1-E25. A comparison between the strengthened slabs and the reference slab reveals a shift from continuous main cracks to discontinuous shorter cracks, attributed to the presence of the composite layer. The transition from wide diagonal cracks to fine, discontinuous cracks is driven by ECC’s multi-stage bridging mechanism. Unlike traditional externally bonded (EBR) FRP systems that often suffer from premature interfacial debonding due to localized stress concentrations [52,53], the ECC matrix distributes strains into multiple micro-cracks via PVA fibers. This deformation compatibility maintains interfacial shear stresses well below the substrate’s cohesive strength, effectively eliminating brittle debonding.

Moreover, the anticipated debonding failure at the interface between the concrete and the composite layer did not occur during testing, further validating the practicality of this composite system. In typical EB-FRP applications, debonding often initiates at flexural cracks and propagates along the FRP–concrete interface, leading to sudden loss of composite action before the FRP reaches its ultimate tensile capacity [54]. By contrast, the ECC matrix in the proposed system exhibits superior bond compatibility with both the concrete substrate and the BFRP grid. The cementitious nature of ECC provides chemical and mechanical interlocking with the roughened concrete surface, while the PVA fibers within the ECC matrix create a three-dimensional fiber network that mechanically anchors the BFRP grid.

### 3.2. Cracking, Yield, and Ultimate Load

**Figure 6:**
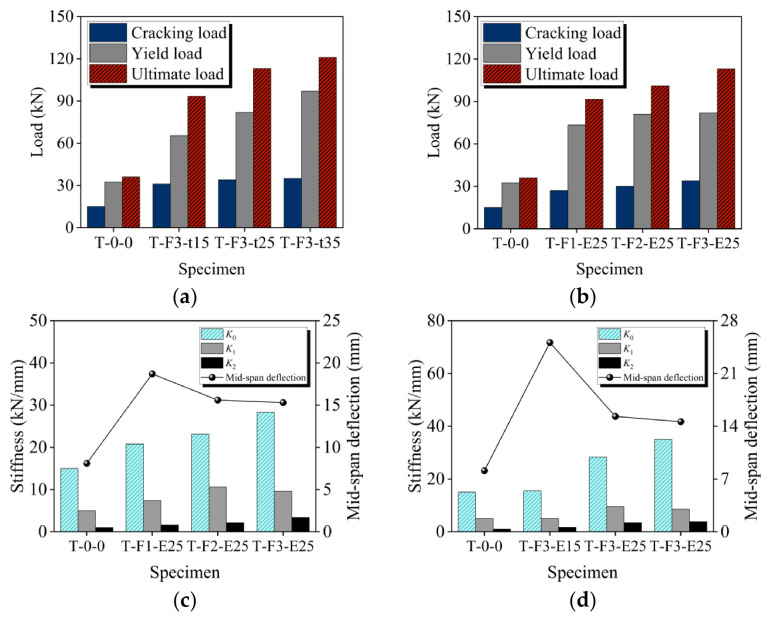
Effect of BFRP and ECC thickness on characteristic loads and stiffness. (**a**) Effect of BFRP thickness on load; (**b**) effect of ECC thickness on load; (**c**) effect of BFRP thickness on stiffness; (**d**) effect of ECC thickness on stiffness.

Figure 6a,b depicts the variations of the cracking load, ultimate load, and maximum mid-span deflection as a function of BFRP grid thickness and ECC thickness, respectively. More detailed data are given in Table 5. Compared to the reference slab, the cracking, yield, and ultimate load of the strengthened slab were significantly increased by 80–133.3%, 126.5–199.7%, and 154.1–236.1%, respectively. Additionally, the augmented mid-span deflection of the reinforced plate, ranging from 80.2% to 209.9%, indicates a remarkable improvement in deformation capacity. This enhancement can be primarily attributed to the superior strain-hardening properties of ECC. It is worth noting that as the thickness of the BFRP grids and ECC increased, the ultimate load of the reinforced plate also increased, while the mid-span deflection gradually decreased. This suggests that a thicker composite layer not only enhances bending capacity, but also mitigates deformation. The increase in load capacity with composite layer thickness follows fundamental flexural mechanics: thicker layers increase the distance from the BFRP grid to the neutral axis, enlarging the moment arm and sectional capacity. However, the concurrent decrease in deflection and material utilization indicates a trade-off between strength and deformation capacity, which is also observed in FRP-strengthened beams [55]. This implies that thicker composite layers are advantageous for strength-critical retrofits, but may be less suitable when ductility is a primary concern.

Table 6 summarizes the enhancement of characteristic load and material utilization based on different reinforcement techniques. It can be observed that the BFRP grid–ECC reinforcement system significantly enhances the characteristic load without the need for complex interface treatments or debonding issues. Additionally, this method eliminates the reliance on epoxy-based adhesives, enhancing its durability.

### 3.3. Load-Deflection Response

**Figure 7:**
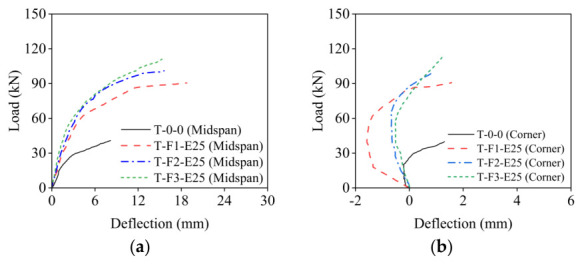

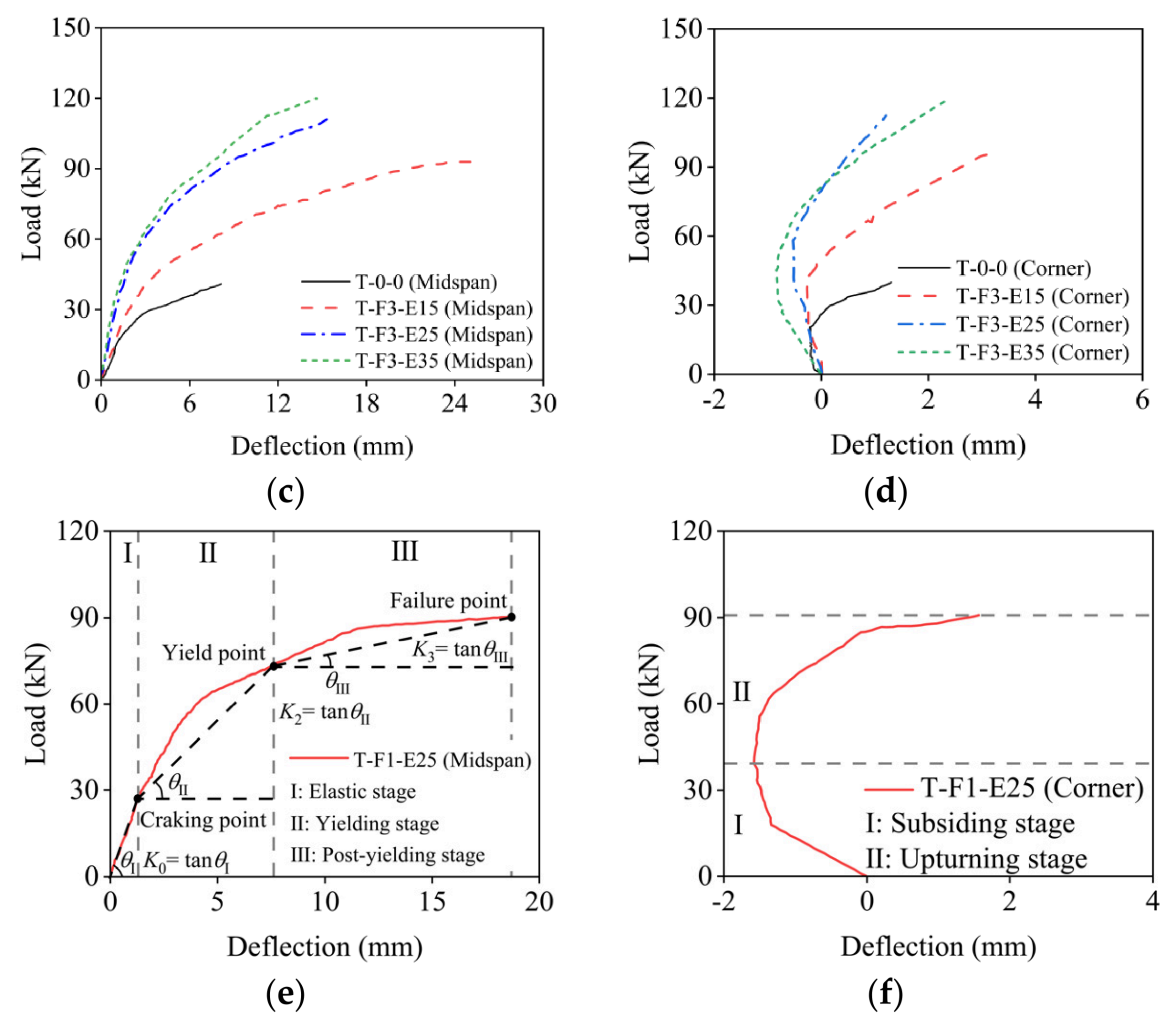
Load-deflection curves at mid-span and corner. (**a**) With different BFRP thickness at mid-span; (**b**) with different BFRP thickness at corner; (**c**) with different ECC thickness at mid-span; (**d**) with different ECC thickness at corner; (**e**) typical load vs. mid-span deflection curve; (**f**) typical load vs. corner deflection curve.

Figure 7a,c presents comparisons of load–mid-span deflection curves for slabs with varying BFRP grid thicknesses and ECC thickness, respectively. The load vs. mid-span deflection curve typically exhibits a three-stage pattern, as depicted in Figure 7e. In the elastic stage, the curve approximates a straight line. Transitioning into the cracking stage, cracks propagate, and the concrete and ECC in the tensile zone gradually cease to bear load, causing a slight decrease in the curve’s slope. Finally, in the yielding stage, reinforcement yields, and significant strain develops in the BFRP, leading to a gradual reduction in the plate’s stiffness until eventual failure. As illustrated in Figure 6c,d, the stiffness at each stage, denoted as *K*_0_, *K*_1_, and *K*_2_, respectively, was found to be higher for strengthened slabs compared to the reference slab, suggesting a deferred rate of stiffness deterioration with the composite layer. Additionally, the ultimate mid-span deflection decreased as BFRP grid and ECC thicknesses increased, attributed to the overall stiffness enhancement. In conclusion, the inclusion of the composite layer improved flexural performance by increasing stiffness.

Figure 7b,d depicts comparisons of load–corner deflection curves for slabs with varying BFRP grid thicknesses and ECC thickness, respectively. The load vs. corner deflection curve typically follows a two-stage pattern, consisting of a subsiding stage and an upturning stage, as illustrated in Figure 7f. In the subsiding stage, the corner deflection aligns with the loading direction. During the upturning stage, as the load approaches approximately 30% of the ultimate load, the corners of the tested slabs gradually begin to lift until failure. The corner lever effect is quantified by the relative deflection *δ*, which is the sum of the maximum positive and negative deflections. The *δ* values for strengthened slabs ranged from 1.61 mm to 3.15 mm, while the control slab had a *δ* of 1.51 mm, indicating a more pronounced corner lever effect in the strengthened slabs. This differential effect arises because the ECC layer primarily enhances flexural stiffness (increasing moment of inertia), whereas the BFRP grid provides in-plane tensile capacity that resists membrane forces induced by corner uplift. A similar corner lifting phenomenon was reported by Deng et al. [64] in CRM-strengthened two-way slabs, attributed to plastic hinge formation along yield lines near corners. Moreover, slabs with thicker ECC displayed larger *δ* values, and increasing the BFRP grid thickness led to a decrease in δ. The corners of the slabs are susceptible to premature fracture due to the corner lever effect, resulting in reduced flexural capacity. Therefore, to address this significant corner lever effect, employing a composite layer with thicker BFRP grid and an appropriate ECC thickness is advisable.

### 3.4. Load–Strain Response of Steel Reinforcement

**Figure 8:**
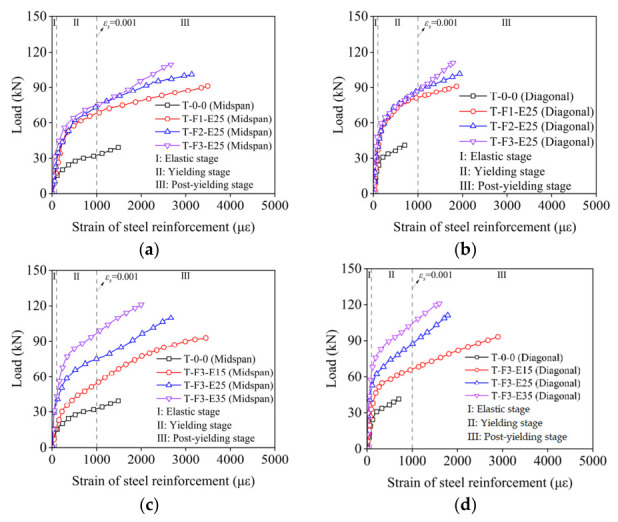
Load–strain curves of steel reinforcement at mid-span and diagonal. (**a**) With different BFRP thickness at mid-span; (**b**) with different BFRP thickness at diagonal; (**c**) with different ECC thickness at mid-span; (**d**) with different ECC thickness at diagonal.

Figure 8 depicts the load–strain curves for steel reinforcement at the mid-span and along the diagonal, showing three distinct stages of response. Initially, an elastic stage is observed where the strain of the steel bar develops gradually. The subsequent stage is marked by the transition from cracking to yielding. The final stage, post-yielding, exhibits a rapid increase in strain. Initially, both steel and BFRP deform elastically; after cracking, the BFRP grid takes up more tension; after steel yielding, the grid becomes the primary tension-resisting component. The strain evolution in steel reinforcement provides insight into load-transfer mechanisms. In reference slab, steel yielding triggers immediate stiffness degradation. In strengthened slabs, the BFRP grid–ECC layer assumes a significant portion of the tensile force after steel yielding, as evidenced by continued load increase beyond the yield point in Figure 8. This load-sharing mechanism, analogous to that observed in FRP grid–ECC beams [36], delays steel strain localization and distributes tensile stresses over a larger area.

The effective utilization of the material is quantified by the utilization rate *λ_s_*, calculated as the maximum strain divided by the ultimate strain. As depicted in Figure 8, the maximum strains of steel reinforcement at mid-span and diagonal for strengthened slab are 3450 με and 2898 με, respectively. It is notable that the maximum strains at mid-span and diagonal for strengthened slabs significantly exceeded those of the reference slab, indicating a substantial enhancement in the *λ_s_* of steel reinforcement in the strengthened slabs. This enhancement can be attributed to the improved mid-span deflection resulting from reinforcement with the BFRP grid–ECC composite, leading to increased deformation of the steel reinforcement.

However, it is important to observe that the *λ_s_* of steel reinforcement declined with the increase in BFRP grid and ECC thicknesses. The decline in steel utilization rate with increasing composite layer thickness can be explained by sectional analysis. A thicker composite layer shifts the neutral axis upward due to additional compressive resistance from the ECC layer. This reduces the lever arm between steel reinforcement and the compressive resultant, requiring less steel strain to achieve moment equilibrium for a given load. Consequently, for the same applied moment, thicker layers produce lower steel strains, reducing utilization. This aligns with ACI 440.2R-17 [66], which notes that increasing FRP reinforcement beyond an optimal value may lead to brittle compression-controlled failure without material efficiency gains.

### 3.5. Load–Strain Response of BFRP Grid

**Figure 9:**
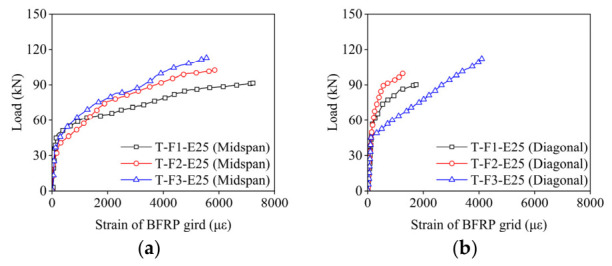

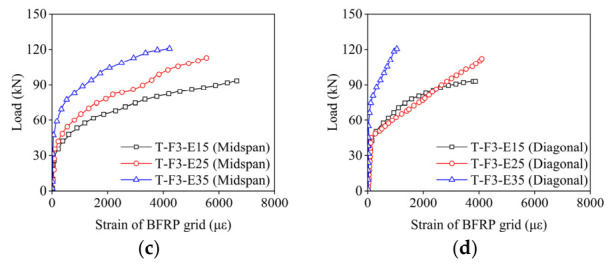
Load–strain curves of BFRP grid at mid-span and diagonal. (**a**) With different BFRP thickness at mid-span; (**b**) with different BFRP thickness at diagonal; (**c**) with different ECC thickness at mid-span; (**d**) with different ECC thickness at diagonal.

Figure 9 displays the strain behavior of the BFRP grid at the mid-span and along the diagonal in the strengthened slab. Initially, the BFRP grid strain is minimal. Upon reinforcement yielding, load is transferred to the BFRP grid, rapidly increasing its strain. Notably, the diagonal BFRP grid consistently exhibits lower strain than the mid-span, indicating higher BFRP grid utilization in the mid-span region. This strain gradient has important design implications: if the BFRP grid were to be optimized for material efficiency, varying the grid density or thickness across the slab (higher reinforcement ratio at the center, lower at the corners) could improve overall material utilization. However, the practical simplicity of uniform grid placement may outweigh marginal efficiency gains for most applications.

The maximum strains of the BFRP grid at the mid-span and diagonal were 7222 με and 3872 με, corresponding to material utilization rates of 31.4% and 16.8%, respectively. As the BFRP grid thickness increased from 1 mm to 3 mm, the strain at the mid-span decreased from 7222 με to 5551 με, with a corresponding reduction in material utilization by 6.7%. Similarly, increasing the ECC grid thickness from 15 mm to 35 mm led to a reduction in BFRP grid strain from 6641 με to 4224 με, with a 10.5% decline in material utilization. This is attributed to the increased flexural rigidity of the composite layer resulting from the greater thickness of the BFRP grid and ECC, which reduces the overall deformation capacity and leads to lower strain levels.

The relatively low utilization rates of the BFRP grid observed in this study are consistent with previous investigations on passive FRP grid strengthening systems [32]. Specifically, its decreasing trend with increasing layer thickness is governed by the interaction between sectional rigidity and the strain distribution mechanism. The addition of thicker BFRP grids and ECC layers significantly enhances the flexural rigidity of the slab. For a given extreme compressive strain in the concrete, a stiffer section results in a smaller curvature and limited overall deformation before failure. Moreover, increasing the thickness of the strengthening layer effectively shifts the neutral axis further toward the tension zone. Based on the plane section assumption, a deeper neutral axis results in a lower tensile strain developed in the BFRP grid for a constant concrete crushing strain. Because the failure of all strengthened specimens was governed by concrete crushing rather than the rupture of the BFRP grid, a significant portion of the BFRP material strength remains untapped. This mismatch between the high strain capacity of the BFRP and the limited deformation capacity of the composite system becomes more pronounced as the reinforcement thickness increases, leading to the observed decline in material efficiency. To improve utilization efficiency in future applications, prestressing of the BFRP grid [67] or optimization of grid configuration [68] may be considered.

## 4. Prediction Model for Flexural Capacity of Strengthened Slabs

### 4.1. Basic Assumptions

The following assumptions are used for the theoretical analysis:

- At all loading stages, the section of the slab satisfies the plane section assumption.
- After cracking, the tensile capacity of the concrete and ECC is neglected. Due to the inherent variability of cracking of cementitious materials, it is considered unsafe to rely on the tensile strength of the matrix in the limit state.
- A linear elastic model is used for the stress–strain response of the BFRP grid.
- When the slab forms a failure mechanism, it is divided into several rigid segments by multiple yield lines, with shear and torsional deformations neglected.
- The rectangular slab is simplified to a square slab, and it is assumed that the reinforcement provided by the steel reinforcement and BFRP grid is the same in both directions.

### 4.2. Bending Moment on the Yield Line

**Figure 10:**
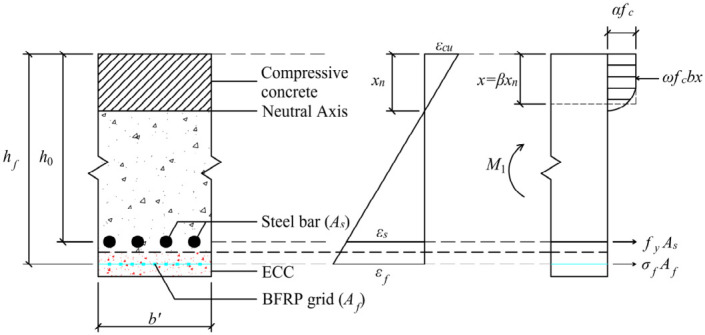
Stresses at cross-section of the strengthened slab.

In Figure 10, the flexural capacity of the strengthened slab is determined by the compressive stress in the concrete, as well as the tensile stresses in the steel reinforcement and BFRP grid. By considering the equilibrium conditions of tensile and compressive stresses in the cross-section, the following formula can be derived:

(1) $$\omega f_{c}b’x=f_{y}A_{s}+\gamma E_{f}\varepsilon_{f}A_{f}$$

Here, *ω* is the equivalent stress reduction factor of concrete in compression zone, *ε_f_* is the effective strain of the BFRP grid, and *A_s_* and *A_f_* are the cross-sectional areas of the tensile steel reinforcement and BFRP grid, respectively. *b*’ indicates the length of the short span. The reduction constant *γ* accounts for the strain inconsistency of the BFRP at different points within the same cross-section, and is commonly taken as 0.4 based on empirical considerations.

In accordance with GB 50608-2020 [69], the bending moment *M*_1_ of the strengthened slab’s cross-section and the height of the compression zone *x* can be expressed as:

(2) $$M_{1}=f_{y}A_{s}(h_{0}-\frac{x}{2})+\gamma E_{f}\varepsilon_{f}A_{f}(h_{f}-\frac{x}{2})$$

(3) $$x=\frac{0.8\varepsilon_{cu}}{\varepsilon_{cu}+\varepsilon_{f}}h_{f}$$

Here, *h*_0_ is the height from the centroid of the reinforcement to the surface of the compressed concrete. *h_f_* is the effective height from the resultant force point of the BFRP grid to the edge of the compressed zone. *ε_cu_* is the ultimate strain of the concrete in the compressive zone.

Following the virtual work method, applying a uniform virtual displacement to loading area C yields an external virtual work *W_e_*, expressed as:

(4) $$W_{e}=\iint \upsilon pdA$$

Here, *υ* denotes the magnitude of vertical virtual displacement, *p* is the load intensity of the loading area, and *dA* is the differential area element of the loading region.

The internal virtual work *W_i_* equals the sum of work done by the bending moments rotating around each yield line, which can be represented as:

(5) $$W_{i}=\sum_{i=1}^{n}\int M_{2}\theta dl_{i}$$

Here, *M*_2_ stands for the bending moment on the yield line, *θ* represents the rotation angle, and *l_i_* is the length of the yield line.

Equating the internal and external virtual work, the load intensity *p* can be defined as:

(6) $$p=\frac{\sum_{i=1}^{n}\int M_{2}\theta dl_{i}}{\iint \upsilon dA}$$

**Figure 11:**
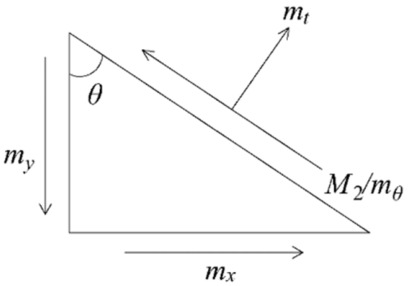
Triangular infinitesimal element.

To determine the ultimate load of the strengthened slab using Equation (6), it is necessary to ascertain the moment on the yield line to compute the internal virtual work of each rigid region. Consequently, a triangular infinitesimal element is selected to investigate the relationship between the moment *M*_2_ on the yield line and the bending moment *M*_1_ of the cross-section, as illustrated in Figure 11. Given *m_x_* and *m_y_* as the ultimate moments per unit length in the *x* and *y* directions, respectively, equilibrium conditions yield the following formulas:

(7) $$M_{2}=m_{\theta }=m_{x}\cos^{2}\theta +m_{y}\sin^{2}\theta$$

(8) $$m_{t}=(m_{x}-m_{y})\sin\theta \cos\theta$$

By substituting $m_{x}=m_{y}$ into Equations (4) and (5), and based on the fifth sub-item of the basic assumption, the expressions simplify to:

(9) $$M_{2}=m_{x}=m_{y}=M_{1}$$

(10) $$m_{t}=0$$

Equation (9) indicates that for strengthened slabs with orthotropic reinforcement, the bending moment per unit width along any direction of the yield line is uniform and equals the ultimate bending moment of the cross-section.

### 4.3. Yield Line Theory

**Figure 12:**
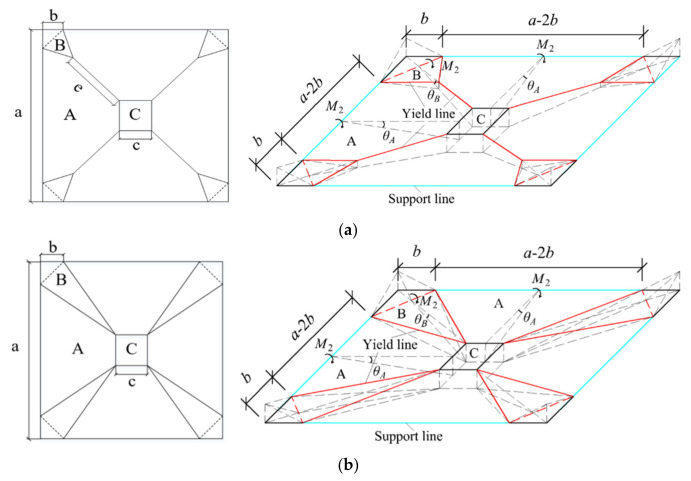

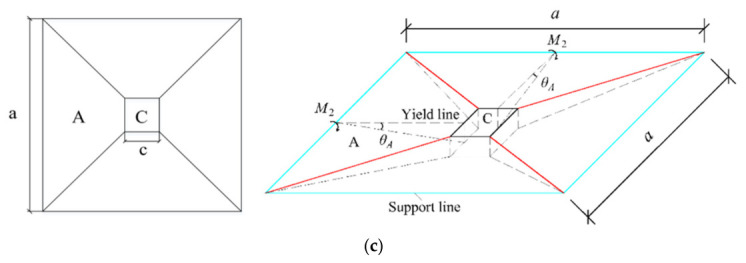
Yield line patterns. (**a**) Y-shaped pattern; (**b**) triangular pattern; (**c**) linear pattern.

After analyzing the crack distribution and the locations of reinforcement yielding, three potential yield line patterns were suggested, as illustrated in Figure 12: the Y-shaped pattern, the triangular pattern, and the linear pattern. In the Y-shaped and triangular patterns, the presence of corner lever effect was taken into account, with corner regions rotating around the axis defined by the dashed line [70].

#### 4.3.1. Y-Shaped Pattern

When considering a unit virtual displacement in the loading area C (refer to Figure 12a), the geometric deformation compatibility conditions lead to the determination of the rotation angles. The rotation angle *θ_A_* of rigid block A around the simply supported edge and the rotation angle *θ_B_* of rigid block B around the dashed axis are expressed as:

(11) $$\theta_{A}=\frac{2}{a-c}$$

(12) $$\theta_{B}=\frac{\sqrt{2}(a-c-\sqrt{2}e)}{\left(a-c)(a-c-b-\sqrt{2}e\right)}$$

Here, *a* denotes the span length of the support, *b* is the horizontal distance from the vertex to the dashed axis, and *e* is the distance from the bifurcation point of the yield line to the vertex of the loading area.

The virtual internal work conducted by rigid blocks A and B, projected onto their respective rotation axes, is represented as *W_A_* and *W_B_*:

(13) $$W_{A}=2M_{1}\frac{a-2b}{a-c}$$

(14) $$W_{B}=2bM_{1}\frac{a-c-\sqrt{2}e}{\left(a-c)(a-c-b-\sqrt{2}e\right)}$$

Consequently, the total virtual internal work *W_i_* of the strengthened slab is given by:

(15) $$W_{i}=4W_{A}+4W_{B}=8M_{1}\left[\frac{a-2b}{a-c}+\frac{b(a-c-\sqrt{2}e)}{\left(a-c)(a-c-b-\sqrt{2}e\right)}\right]$$

Following the principle of virtual work, the external work equals the internal work. As a result, the ultimate load *P_u_*_1_ of the strengthened slab can be determined as:

(16) $$P_{u1}=8M_{1}\left[\frac{a-2b}{a-c}+\frac{b(a-c-\sqrt{2}e)}{\left(a-c)(a-c-b-\sqrt{2}e\right)}\right]$$

#### 4.3.2. Triangular Pattern

Equation (16) reveals that the ultimate load-carrying capacity of the Y-shaped mode changes with variations in *b* and *e*. The solution derived from the plastic hinge theory represents an upper bound solution. To determine the minimum value of the solution outlined in Equation (16), the first derivatives with respect to *b* and *e* are taken:

(17) $$\frac{dP_{u1}}{db}=8M_{1}\left[\frac{{\left(a-c-\sqrt{2}e\right)}^{2}}{(a-c){\left(a-c-b-\sqrt{2}e\right)}^{2}}-\frac{2}{a-c}\right]$$

(18) $$\frac{dP_{u1}}{de}=\frac{8\sqrt{2}M_{1}b^{2}}{(a-c){\left(a-c-b-\sqrt{2}e\right)}^{2}}$$

Based on Equation (18), P*_u_*_1_ increases with an increase in *e*. Considering that $\frac{a-c-\sqrt{2}e}{2}>b>0$ and $\frac{\sqrt{2}}{2}(a-c)>e\geq 0$, it is evident that $\frac{dP_{u1}}{de}>0$. Consequently, when e=0 and $b=\frac{\left(2-\sqrt{2})(a-c-\sqrt{e}\right)}{2}$, *P_u_*_1_ can achieve its minimum value, corresponding to the triangular mode depicted in Figure 12b. By substituting e=0 and $b=\frac{\left(2-\sqrt{2})(a-c-\sqrt{e}\right)}{2}$ into Equation (16), the ultimate load-carrying capacity of the strengthened slab in the triangular mode is determined as:

(19) $$P_{u2}=8M_{1}\left[\frac{a}{a-c}+2\sqrt{2}-3\right]$$

#### 4.3.3. Linear Pattern

From Equations (17) and (18), it is also evident that if b=0, then $\frac{dP_{u1}}{de}=0$. When $e\to \frac{\sqrt{2}}{2}(a-c)$, the yield line approaches a linear mode. In this scenario, rigid plate B is absent, and the positive plastic hinge extends to the corners of the slab. By substituting b=0 and $e=\frac{\sqrt{2}}{2}(a-c)$ into Equations (15) and (16), the following expressions can be derived:

(20) $$W_{i}=W_{A}=2M_{1}\frac{a}{a-c}$$

(21) $$P_{u3}=8M_{1}\frac{a}{a-c}$$

### 4.4. Validation of Formula

**Figure 13:**
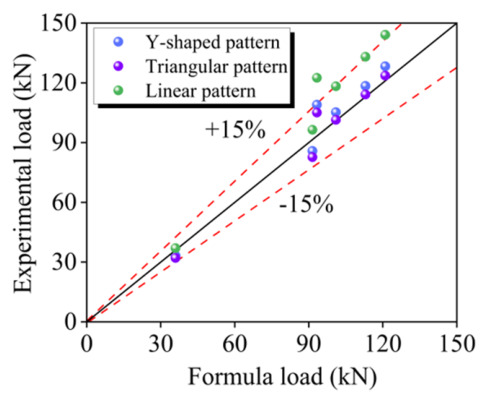
Comparison between predicted results and test results.

The validity of the proposed ultimate load formulas was confirmed by comparing test results with predicted results. In Table 7, it is evident that for all three patterns (Y-shaped, triangular, and linear), the average ratios of calculated ultimate loads to the experimental values are 1.03, 0.993, and 1.15, respectively, with coefficients of variations (COVs) of 0.079, 0.079, and 0.081. The majority of the predictions were notably accurate, with deviation below 15%, as depicted in Figure 13.

It is noteworthy that the results of Y-shaped and triangular patterns demonstrated higher accuracy compared to the linear pattern. This can be attributed to the consideration of the corner lever effect in the Y-shaped and triangular patterns, leading to a reduction in the ultimate load. These results signify that the formulas effectively predict the flexural bearing capacity of the strengthened slab.

However, it is important to mention that the current study’s prediction models are specifically suitable for strengthened slabs with a single *a*/*c* ratio. Future investigations will be essential to further validate the proposed formulas across various *a*/*c* ratios. The theoretical derivation assumes perfect bond at the old concrete–ECC interface until ultimate failure, which aligns with our static experimental observations, but may need modification if applied under fatigue or cyclic loading where interface degradation could occur.

## 5. Conclusions

This investigation into the flexural performance of BFRP grid–ECC composite reinforced two-way RC slabs involved an unreinforced reference slab and five strengthened slabs. Combining experimental findings with theoretical analysis, the following key conclusions emerge:

1. The flexural performance of the strengthened slabs saw significant enhancement. Cracking load, yielding load, and ultimate loads increased by 80–133.3%, 126.5–199.7%, and 154.1–236.1%, respectively. The cracking pattern shifted from diagonally concentrated through-length cracks to discontinuous short cracks. Moreover, the absence of debonding between ECC and the original concrete confirms the method’s feasibility.
2. The impacts of two variables on characteristic load, stiffness, and strain response were meticulously examined. Experimental results revealed that characteristic load and representative stiffness increased with BFRP grid and ECC thicknesses, albeit with some loss in ductility. Initially, a higher utilization rate of steel bars was achieved with the composite layer. However, as BFRP grid and ECC thicknesses increased, utilization rates for both steel bars and BFRP grid gradually declined.
3. Three yield line patterns were introduced to predict the flexural capacity of the strengthened slab. The Y-shaped and triangular models, considering the corner lever effect, yielded results more consistent with experimental data, serving as valuable references for practical engineering applications. Further experimental tests are necessary to validate these proposed models.
4. The demonstrated improvements in ductility and the shift toward fine, discontinuous cracking patterns offer a reliable technical basis for reinforcing aging infrastructure, particularly where clearance constraints and fire safety (due to the inorganic matrix) are critical.
5. This study primarily focused on the static flexural response under specific span-to-loading area ratios. Future research should evaluate the long-term durability of the interfacial bond under environmental stressors (e.g., freeze–thaw cycles or chloride attack) and investigate the structural reliability under fatigue or dynamic loading to facilitate broader engineering applications.

## Figures and Tables

**Table 1:** Details of specimen geometry.

Specimen	*h* (mm)	*h_f_* (mm)	*h_e_* (mm)	*S_s_* (mm^2^)	*S_f_* (mm^2^)
T-0-0	80	/	/	1800 × 1500	1600 × 1300
T-F1-E25	90	1	25	1800 × 1500	1600 × 1300
T-F2-E25	90	2	25	1800 × 1500	1600 × 1300
T-F3-E25	90	3	25	1800 × 1500	1600 × 1300
T-F3-E15	80	3	15	1800 × 1500	1600 × 1300
T-F3-E35	100	3	35	1800 × 1500	1600 × 1300

Note: *h*, *h_f_*, and *h_e_* are, respectively, the height of specimen, BFRP grid, and ECC; *S_s_* and S*_f_* are the size of steel mesh and BFRP grid, respectively.

**Table 2:** Material performance data statistics.

Matreial	Index	Sample 1 (Mpa)	Sample 2 (Mpa)	Sample 3 (Mpa)	Mean (Mpa)	SD (Mpa)	COV (%)
Concrete	*f_c_*_u_	28.07	25.48	25.36	26.3	1.25	4.75
ECC	*f_e,c_*_u_	37.3	38.75	35.64	37.23	1.27	3.41
	*f_e,tu_*	3.6	3.8	3.91	3.77	0.13	3.40
Steel rebar	*f_y_*	352	348	359	353	4.55	1.29
	*f_u_*	509	511	523	514.3	6.18	1.20
BFRP grid (1 mm)	*f_fu_*	2415	2422	2426	2421	4.55	0.19
BFRP grid (2 mm)		2358	2363	2365	2362	2.94	0.13
BFRP grid (3 mm)		2417	2423	2426	2422	3.74	0.15

Note: *f_c_*_u_ and *f_e,c_*_u_ are, respectively, the 28-day compressive strength of the concrete and ECC sample; *f_e,tu_* is the 28-day ultimate tensile strength of the ECC sample; *f_y_* and *f_u_* are, respectively, the yield strength and ultimate tensile strength of the rebar sample; *f_fu_* is the ultimate tensile strength of the single BFRP strip sample.

**Table 3:** Properties of BFRP grid.

No.	*h_f_* (mm)	*A_f_* (mm^2^)	*E_f_* (GPa)	Elongation (%)
B1	1	5	86	2.4
B2	2	10	87	2.3
B3	3	15	85	2.3

Note: *E_f_* is the elastic modulus of BFRP; *A_f_* is the cross-sectional area of BFRP grid; B1 represents BFRP grid with a thickness of 1 mm.

**Table 4:** Material consumption of test specimens.

Specimen	Total Mass (kg)	Concrete (kg)	Steel Rebar (kg)	ECC (kg)	BFRP (kg)
T-0-0	523.95	515.96	7.99	/	/
T-F1-E25	573.15	455.96	7.99	108.33	0.87
T-F2-E25	573.15	455.96	7.99	107.45	1.75
T-F3-E25	573.15	455.96	7.99	106.58	2.62
T-F3-E15	514.59	441.08	7.99	62.9	2.62
T-F3-E35	631.71	470.84	7.99	150.26	2.62

**Table 5:** Summary of experimental results.

ID	Failure Mode	*P_c_* (kN)	*d_c_* (mm)	*P_y_* (kN)	*d_y_* (mm)	*P_u_* (kN)	*d_u_* (mm)	*K*_0_ (kN/mm)	*K*_1_ (kN/mm)	*K*_2_ (kN/mm)	*μ*	*E_n_* (J)
T-0-0	CC	15	1	32.4	4.5	36	8.1	15	5	1	1.8	231.28
T-F1-E25	CE	27	1.3	73.4	7.6	91.5	18.7	20.8	7.4	1.6	2.46	1335.33
T-F2-E25	CE	30	1.3	80.9	6.1	101	15.6	23.1	10.6	2.1	2.56	1201.96
T-F3-E25	CE	34	1.2	81.9	6.2	113	15.3	28.3	9.6	3.4	2.47	1236.92
T-F3-E15	CE	31	2	65.4	8.9	93.3	25.1	15.5	5	1.7	2.82	1726.15
T-F3-E35	CE	35	1	97.1	8.3	121	14.6	35	8.5	3.8	1.76	1263.48

Note: *P_c_*, *P_y_*, and *P_u_* are the cracking, yield, and ultimate load, respectively. *d_c_*, *d_y_*, and *d_u_* are the corresponding mid-span deflection at the cracking, yield, and ultimate load, respectively. CC and CE represent concrete cracking and ECC cracking, respectively. *K*_0_, *K*_1_, and *K*_2_ are the representative stiffness at different loading stages. *K*_0_ = *P_c_*/*d_c_*, *K*_1_ = (*P_y_* − *P_c_*)/(*d_y_* − *d_c_*), *K*_2_ = (*P_u_* − *P_y_*)/(*d_u_* − *d_y_*). *μ* is ductility index. *E_n_* is the energy absorption capacity.

**Table 6:** Data comparison.

Researcher	Strengthening Technique	Subject	Dimension (mm)	Anchor/Interface Configuration	Debonding	∆*P_c_* (%)	∆*P_y_* (%)	∆*P_u_* (%)	*λ_s_* (%)	*λ_f_* (%)
Authors’ study	BFRP grid–ECC	Two-way slab	1800 × 1500 × 80	Chiseling	No debonding	80~133.3	101.9~199.7	154.1~236.1	15~35	4.5~31.4
Zheng et al. [50]	BFRP grid–ECC	Beam	1800 × 200 × 300	Chiseling	Partial debonding after the rupture of BFRP grid	58.1~96.8	14.9~34.7	4~32.5	50~70	27~46
Yang et al. [34]	CFRP grid–ECC	Beam	2000 × 200 × 150	U warp anchor/Chiseling/Epoxy resin adhesive	Critical diagonal/Intermediate crack-induced debonding	87.5–425	26.7–102.9	35.5–116.8	24~73	24~96
Wu et al. [33]	CFRP grid–ECC	Beam	2300 × 250 × 150	Steel reinforcement wrapped by ECC	No debonding	20~100	5.9~16	−7~20	25~30	/
Zhou et al. [32]	EB CFRP strips	One-way slab	3200 × 250 × 150	Hybrid anchor/Polishing	Intermediate crack-induced debonding	0~14.3	13~21.7	−3.3~10	/	42~87
Torabian et al. [56]	EB\EBORG CFRP sheets	Two-way slab	2200 × 2200 × 150	Epoxy resin adhesive	Partial debonding	23.5~35.7	/	2~39	2.5~30	0~50
Moshiri et al. [57]	EB\EBORG CFRP strips	One-way slab	6000 × 1000 × 220	End anchor system/Epoxy resin adhesive	Intermediate crack-induced debonding	/	/	32~35	/	45~78
Codina et al. [58]	EB\HB CFRP laminates	Beam	2400 × 180 × 140	Epoxy resin adhesive	Intermediate crack-induced debonding	/	/	8~27	/	39~77
Hosseini et al. [59]	NSM CFRP laminates	One-way slab	2600 × 600 × 120	Epoxy resin adhesive	No debonding	0~371.8	19.8~119.4	58.6~134	/	13~96
Aljidda et al. [60]	NSM BFRP/GFRP bars	One-way slab	3000 × 600 × 150	Epoxy resin adhesive	No debonding	12.5~77.8	40.4~71.8	82.4~101.6	/	65~69
Dias et al. [61]	NSM CFRP laminates	Beam	2400 × 150 × 300	Epoxy resin adhesive	Intermediate crack-induced debonding	4.5~19.4	10.2~34.3	41.9~103.2	/	83~100
Su et al. [62]	Prestressed NSM CFRP strips	Beam	2700 × 150 × 250	Epoxy resin adhesive	End interfacial debonding + End cover separation	−233.3	/	−12.3~136.8	10–50	11–62
Zhang et al. [63]	TRM	One-way slab	4200 × 600 × 100	Geopolymer mortar or Polymer modified cement mortar/ grinding and cleaning	No debonding	/	/	12~92	30–98	15.9–30.5
Deng et al. [64]	TRM	Two-way slab	1800 × 1300 × 100	Epoxy resin adhesive	No debonding	123.4~191.2	/	43.8~54.2	/	/
Koutas and Papakonstantinou [65]	TRM	Beam	1600 × 120 × 200	Fiber-reinforced cement-based mortar with synthetic \polymers	Intermediate crack-induced debonding	/	/	7.4–37.4	/	/

Note: ∆*P_c_*, ∆*P_y_*, and ∆*P_u_* are increase of the cracking, yield, and ultimate load, respectively; *λ_s_* and *λ_f_* are, respectively, the maximum utilization rate of steel reinforcement and BFRP grid.

**Table 7:** Comparison of experimental and calculated results.

ID	*P*_u_ (kN)	Y-Shaped Pattern		Triangular Pattern		Linear Pattern	
		*P*_u1_ (kN)	*P*_u1_/*P*_u_	*P*_u2_ (kN)	*P*_u2_/*P*_u_	*P*_u3_ (kN)	*P*_u3_/*P*_u_
T-0-0	36	33.29	0.832	32.08	0.891	37.04	1.028
T-F1-E25	91.5	85.82	0.938	82.71	0.904	96.42	1.054
T-F2-E25	101	105.27	1.042	101.44	1.004	118.27	1.171
T-F3-E25	113	118.47	1.048	114.17	1.010	133.10	1.179
T-F3-E15	93.3	109.05	1.168	105.09	1.126	122.52	1.313
T-F3-E35	121	128.29	1.060	123.63	1.022	144.13	1.191
Average	/	/	1.030	/	0.993	/	1.150
COV	/	/	0.079	/	0.079	/	0.081

Note: *P_u_*_1_, *P_u_*_2_, and *P_u_*_3_ are the calculated ultimate load from Y-shaped, triangular, and linear pattern, respectively.

## Data Availability

The original contributions presented in this study are included in the article. Further inquiries can be directed to the corresponding author.

## Abbreviations

The following abbreviations are used in this manuscript:

RC	Reinforced concrete
BFRP	Basalt fiber-reinforced polymer
ECC	Engineered cementitious composite
EB	Externally bonding
NSM	Near-surface-mounted
CRM	Composite-reinforced mortar
PVA	Polyvinyl alcohol
LVDT	Linear variable differential transformer
SG	Strain gauge
